# Supplementary material for: Cellular and Molecular Mechanisms Mediating Methylmercury Neurotoxicity and Neuroinflammation
Source: Int J Mol Sci. 2021 Mar 18;22(6):3101. doi: 10.3390/ijms22063101 (PMC8003103; doi:10.3390/ijms22063101)
Supplement: Supplementary file 1 [file ijms-22-03101-s001.pdf]

Microglia			
**Biological Model**	**MeHg exposure protocol**	**Cellular alterations**	**Reference**
Rat microglial primary culture	0.5; 5 μM MeHg for 1, 2, 3, and 4 days	↑Microglial cell death; ↑Cas-3 activation; ↑Round shape; Inhibition of Cas-3 activity decreased cell death	[1]
Rat microglial primary culture	0.0001-1 μM MeHg for 5 or 10 days	↑Process retraction, round-shape, and cell body; ↑Clustering; ↑Cell death; ↓IL-6, no changes in TNF-α; no changes in NO	[2]
N9 mouse microglial cell line	5-40 μM MeHg for 30 min-24h	↑ROS; ↑Mitochondrial depolarization; ↓Aconitase activity and cytoplasmic content; ↑Il-6; 15d-PGJ_2 attenuates MeHg-induced cell death	[3]
N9 mouse microglial cell line	2 ng/mL; 2 μg/mL MeHg for 48h	↑Cell viability; ↑Number of microglia; ↓TNF-α; ↓IL-1β; ↓TNF-α mRNA; no changes in IL-1β; ↑NO; ↑Ca^{2+} (2 ng/mL MeHg)<br>↓Cell viability; ↓Number of microglia; ↑TNF-α; ↑IL-1β; ↑TNF-α mRNA; ↑IL-1β mRNA; ↓NO; ↓Ca^{2+} (2 μg/mL MeHg)	[4]
Mouse microglial primary culture	0.1; 0.5; 1 μM MeHg for 3 days + PAM (3) for 2 days	↓Cell viability; ↓IL-6; no changes in TNF-α	[5]
Rat microglial primary culture	0.1; 1; 5 μM MeHg for 1 min-6h	↓Cell viability; ↑ROS; ↓GSH/GSSG ratio; ↑Nrf2 as a protective response	[6]
Rat microglial primary culture	0.1; 1; 5 μM MeHg for 1 min-6h	Microglia is more sensitive than astrocytes (↓cell viability, ↑ROS, ↓GSH/GSSG ratio; ↑Nrf2 in earlier timepoints)	[7]
Rat aggregating brain cell cultures	0.001-1 μM MeHg Days 5-15 and 25-35	No death of microglia nor clusters surrounding apoptotic cells	[8]
C6 rat glioma cell line<br>U251HF human glioma cell line	2.5-10 μM MeHg for 30 min-16 h	↑ROS (in C6 glioma cells); ↑IL-6 (C6 and U251HF glioma cells)	[9]
Mouse Organotypic cortical slices<br>Mouse microglial primary culture	0.1 μM MeHg for 1 or 21 days renewed every 2/3 days (slices)<br>24 or 96 h (primary cultures)	↓Process number; ↓Length; ↑Body size; ↑Sholl alterations; ↓process extension and retraction; ↑Staining of microglia; ↑iNOS, ↑TNF-α, ↑ROCK; ↑P-MYPT1(organotypic cortical slices)<br>↑ROCK; ↑P-MYPT1; ↑TNF-α; ↑iNOS; ↑circularity; ↓Process number (primary cultures)	[10]
Mouse Organotypic cerebral cortex slices	1; 5 μM MeHg 8h	↑CD16 mRNA; ↑CD32 mRNA; ↑Amoeboid microglia; ↓Process number	[11]
Adult *Wistar* rats (8 weeks)	5 mg/kg/day MeHg for 12 days + 8 days free of MeHg	↑Reactive microglia and ↑Activated morphology accumulated in the granular cell layer of the cerebellum; ↑Cathepsin B colocalized with reactive microglia	[12]
C57BL/6J Mice (gestation days 6-9)<br>Hippocampal sandwich cultures	1;10 mg/Kg/day MeHg C57BL/6J Mice (gestation days 6-9)<br>1,33 nM MeHg Hippocampal sandwich cultures for 3 days	↑Microglia reactivity (mice hippocampus and hippocampal sandwich cultures)<br>↑Process thickness; ↑Circularity; ↓Cell area; ↓Process number (hippocampal sandwich cultures)	[13]
7 weeks old *Sprague-Dawley* rats	1 mg/Kg/day MeHg for 1,2,3 or 4 weeks + 12 weeks free of MeHg	↑Staining of microglia in the dorsal root nerve and the dorsal column of the spinal cord;<br>↑TNF-α; ↑iNOS; ↑IL-1β; ↑IL-6; ↑p-NF-kBp65; ↑ROCK	[14]

Adult female monkeys	50 μg/Kg/day MeHg for 6,12 and 18 months	↑Reactive glia in the cortex of the calcarine sulcus (visual cortex)	[15]
Adult female monkeys	50 μg/Kg/day MeHg for 6,12 and 18 months	↑Number of microglia; ↑Mercury inside microglia relative to other cells; ↑Mercury inside microglia earlier than other cells (except astrocytes) in the cortex of the calcarine sulcus	[16]
Rat aggregating brain cell cultures	0.001-1 μM MeHg Days 5-15 and 25-35	↑Clustering and ↑Number of microglia	[17]
Adult female common marmosets	1.5 mg Hg/kg/day MeHg for 2 weeks	↑Staining and ↑Number of microglia in the occipital lobes	[18]
6 weeks old male C57BL/6NJcl mice	1.5 mg/Kg/day MeHg for 8 weeks	↑Staining of microglia in the motor and prelimbic cortex	[19]
Adult male *Wistar* rats	6.7 mg/Kg/day MeHg for 5 days + 2 days free of MeHg (for day 7 tissue samples). Cycle repeated once for day 14 samples	↑Staining (dorsal root ganglions and sensory fibres) ↑Number of microglia aggregates (dorsal root ganglions)	[20]
8 weeks old C57BL/6 male mice	Single 25 mg/Kg MeHg administration + 5 or 7 days free of MeHg	↑TNF-α mRNA in cerebrum and cerebellum	[21]
Rat microglial primary culture Mouse microglial primary culture	0.01 – 3 μM MeHg for 2-24h	↑ATP release; ↑p38 MAPK phosphorylation; no changes in IL-6	[22]

Table S1: Biological models and MeHg exposure protocols used to evaluate microglial cellular alterations.

Oligodendrocytes			
**Biological Model**	**MeHg exposure protocol**	**Cellular alterations**	**Reference**
Adult female monkeys	50 µg/Kg/day MeHg for 6,12 and 18 months	↑Scattered oligodendrocytes with Hg deposits	[15]
Adult female monkeys	50 µg/Kg/day MeHg for 6,12 and 18 months	No significant change in total number of oligodendrocytes	[16]
Rat aggregating brain cell cultures	0.001-1 µM MeHg Days 5-15 and 25-35	↓2',3'-Cyclic-nucleotide 3'-phosphodiesterase enzymatic activity	[17]
Human MO3.13 oligodendroglia cell line	10-100 µM MeHg for 24h	↓Cell viability	[23]
Human *post-mortem* brain	50 individuals with *pre-mortem* medical conditions including Hg self-injection	↑Accumulation of Hg inside oligodendrocytes (geniculate nuclei)	[24]
Human *post-mortem* brain	Self-injected intravenously with metallic Hg taken from thermometers	↑Accumulation of Hg inside oligodendrocytes (cerebral cortex)	[25]

Table S2: Biological models and MeHg exposure protocols used to evaluate oligodendrocytic cellular alterations

Astrocytes			
**Biological Model**	**MeHg exposure protocol**	**Cellular alterations**	**Reference**
Wistar adult rats	5 mg/kg/day MeHg for 2 weeks	↑S100β in CSF	[26]
Mouse astrocytic primary culture	1; 3 μM MeHg for 12h and 24h	↑IL-6 mediated neuroprotection via ATP/P2Y1 receptor	[27]
Mouse astrocytic primary culture	0.1; 0.5; 1 μM MeHg for 3 days + PAM (3) for 2 days	No alterations in IL-6 and TNF-α in astrocytes	[5]
Rat neonatal primary astrocytes	2.5, 5, 10, and 20 μM MeHg for 6-30 h	↑ Apoptosis; ↑ LDH release; ↑ Oxidative stress; ↑ROS; ↓ GS activity; ↓GLAST mRNA; ↓GLT-1 mRNA; ↓Cell density; ↑Cell shrinkage; ↑Cavitation changes	[28]
Rat neonatal primary astrocytes	1; 5; 10 μM MeHg for 1-24h	↓[(3)H]-glutamine uptake; ↓Mitochondrial potential; ↑ERK phosphorylation;↑Caspase-3 activation	[29]
Rat neonatal primary astrocytes	5;10 μM MeHg for 1 or 6 h	↑Lipid peroxidation; ↑ ROS; ↓Mitochondrial potential; ↓[(3)H]-glutamine uptake	[30]
Rat neonatal primary astrocytes	1-10 μM MeHg for 60 min	↓Cys uptake via Na(+)-dependent cys transporters ASC and X(AG(-))system	[31]
Rat neonatal primary astrocytes	1−10μM MeHg for 30 minutes	↑ Morphological alterations; ↑GSH impairment; ↑ROS; ↓Cell proliferation; ↓ HIF-1α mRNA	[32]
Rat neonatal primary astrocytes	1-3 μM MeHg for 24h	↓ Cell viability; No apoptotic-induced markers; ↑Necrosis; ↑JNK activity; ↑TNF-α release; Fusiform morphology; ↑GLAST; ↑ROS; ↑SOD-1 activity; ↑CAT activity, ↓GPx; ↓Sulfhydryl content	[33]
Rat neonatal primary astrocytes	0.01-10 μM MeHg for 6h	↓GSH; ↓GSSG; ↑Nrf2 activity; ↑Nrf2 nuclear translocation; ↑HO-1 mRNA; ↑NQR mRNA	[34]
Rat neonatal primary astrocytes	5 μM MeHg for 6 h	↑Oxidative Stress; ↑ROS; ↓GSH; ↓catalase levels	[35]
Mouse astrocytic primary culture	5 μM MeHg for 30 min	↑Oxidative Stress; ↑ROS; ↓GSH	[36]
CCF-STTG1 human astrocytoma cell line	1-10 μM MeHg for 24h	↑ GSSG; ↑GSH/GSSG; ↑Total glutathione (GSH+GSSG); ↑GPx, ↑Glutathione synthetase; ↑Nrf2; Changes in S-glutathionylation patterns	[37]
Mouse astrocytic primary culture	10 μM MeHg for 6h	↓Cell viability; ↓ ATP; ↑LDH; ↑ROS; ↓ NAD^+/NADH; ↑Cas-9; ↑Cleaved Cas-3; ↑ROCK, ↑ROCK downstream signalling	[38]
Adult male *Wistar* rats	0.04 mg/kg/day MeHg for 60 days<br>By gavage	Hg deposits in the visual cortex;<br>↓NADPH diaphorase neuropil reactivity; Astrocytic morphological alterations (hypertrophic and swelled cell bodies; shorter and thicker processes); ↓Astrocytic number	[39]

Table S3: Biological models and MeHg exposure protocols used to evaluate astrocytic cellular alterations

Neurons			
**Biological Model**	**MeHg exposure protocol**	**Cellular alterations**	**Reference**
Sprague-Dawley rats and Rat primary CGC	0.1 - 30µg/g of MeHg for 7h, 24h and 2 weeks; 0.1-10µM MeHg for 6h and 24h	*In vivo*:↓ DNA synthesis in hippocampus<br>*In vitro*:↓ DNA synthesis ↓ S-phase entry<br>↓ pro-mitogenic cyclin E<br>↑Cleaved Cas-3	[40]
Sprague Dayley rats	0,2-5 µg/g MeHg (sci) for 24h or 2weeks	↓ Immature DCX⁺ cells in DG<br>↑ Apoptosis of proliferative NSC in hilus and GCL<br>↓ Cell number in hilus and GCL<br>Hippocampal-dependent memory deficits	[41]
Sprague–Dawley rats	2.5–10 µg/gbw MeHg for 8 and 24 h<br>1.5 µmol/L of MeHg for 24h	In vivo:↓ DNA synthesis in hippocampus<br>Changes in DG structure<br>↓ cells in GCL and hilus<br>↓ cyclin E; cyclin D1 and D3<br>↑Cleaved Cas-3 ↑oxidative stress via ROS<br>↓ hippocampal-dependent memory<br><br>In vitro cortical cells: ↓ DNA synthesis ↓ cyclin E<br>↑Cleaved Cas-3	[42]
Sprague–Dawley rats	0.6µg/g or 5µg/g MeHg for 24h, 14 days (sci)	↓ Sox2 cells in hilus and CGL	[43]
Rat Primary hippocampal NSC	100 pmol/L-10 nmol/L MeHg for 48h	↓ DCX<br>↓Neurogenic differentiation MAP-2 expression<br>↑ Astrogliogenic differentiation GFAP	[44]
Human SH-SY5Y neuroblastoma cell line and mouse cortical NPC	SH-SY5Y: 0.1-10 µM MeHg for 5h<br>NPC: 0.03-3 µM MeHg for 5h	Low doses promote ↑ CNTF-evoked P-STAT3<br>↑ CNTF-evoked GFAP in NPC<br>High doses promote oxidative stress via ROS	[45]
Wister rats	0.04 mg/kg/day MeHg in gavage for 60 days	Impairment of cognitive functions<br>↓ NeuN⁺ cells in hippocampus<br>↓ Antioxidant capacity	[46]
Wister rats	0.04 mg/kg/day MeHg in gavage for 60 days	↓ NeuN⁺ cells in motor cortex<br>↓ Antioxidant defense<br>↑ Nitrite levels<br>↑ Lipid peroxidation	[47]
Adult male *Wistar* rats	6.7 mg/Kg/day MeHg for 5 days + 2 days free of MeHg (for day 7 tissue samples). Cycle repeated once for day 14 samples	↑ Neural cell death<br>↓NeuN cells ↓axons in sensory neurons<br>↓Neurofilament in sensory fibers (axonal marker)	[20]
Rat PC12 differentiating neuronal cell line and primary CGC	1 nM–100 µM MeHg	↓ Neurite outgrowth	[48]
Sprague–Dawley rats	4; 8 mg/kg MeHg on gestation	Primary cultures of pup cortical neurons:↓ cell viability; ↑apoptosis<br>↑glutamate-induced apoptosis<br>↓ MAP2 immunoreactivity<br>↓ β3-tubulin immunoreactivity	[49]
Wistar rats	0.05; 0.25 mg/kg/day MeHg	↓ NF-H<br>↓Presynaptic proteins: SPP; SNAP25<br>↓ Post synaptic proteins: PSD-95	[50]

Rat PC12 differentiating neuronal cell line	100 nM MeHg for 24h	↓ NF-H<br>↑ neuronal cell death by apoptosis<br>Inhibition of neurites extension	[51]
Wistar rats	1.5 mg/kg MeHg<br>GD5 - partiture	Alterations in synaptic plasticity and neurotransmission in the developing rat brain; ↓BDNF; ↓GDNF; ↑GFAP in hippocampus	[52]
Human neural progenitor cells ReNcell CX cell line	10; 50 nM MeHg for 24h	↓ Mitochondrial metabolic function; ↑ apoptosis; ↑ ROS production;<br>↓Mitochondrial potential<br>↓ATP produced	[53]
Mouse Neuro-2a cell line	1-5 µM MeHg for 24h	↑Apoptotic cell death (activation of PARP; PS exposure and ↑Cleaved Cas-3); ↑ER stress and ↑ROS-mediated Akt inactivation	[54]
C.Elegans	0.05; 0.5; and 5 µM of MeHg	Changes in cephalic dopaminergic neurons	[55]
Human SH-SY5Y cell line	50 nM MeHg for 24h	↓Cell viability; ↓Glutamate-mediated cell viability via NMDA receptor	[56]
Sprague–Dawley rats	5mg/kg MeHg gavaged for 7days	Changes in mRNA of NMDA receptor subunits in hippocampus and cerebral cortex: ↓NR2A; ↓NR2B (hippocampus) and ↑NR2C	[57]
hiPSC-MNs	0.1; 0.2; 0.5; 1 and 1.5 µM MeHg for 1h (+24h recovery) or 24h	Biphasic increase in Ca^{2+} mediated by AMPA/KA receptor; Fragmentation of neurites	[58]
Spinal cord slices from the lumbar region of young adult male mice	20 µM MeHg for 5-25 min	↑Neurotoxicity and hyperexcitability; ↑Ca^{2+}	[59]
Rat Primary Cerebellar Granular Neurons	100 nM MeHg for 24h or 48h	↓Cell viability; ↓BDNF-mediated cell viability; ↓GSH	[60]
Human SH-SY5Y neural cell line	0.03-9 µM MeHg for 72h	↑Cell death (DNA oxidative damage; Cas-1; Cas-3 and Cas-8); ↑Pro-inflammatory cytokines (IL-1β; IL-6; TNF-α; IFNγ) and ↓Anti-inflammatory cytokines (IL-10)	[61]
Human SH-SY5Y neural cell line	250-1250 nM MeHg for 24h	↓Differentiated and non-differentiated cell viability; ↑Oxidative stress in differentiated cells	[62]
Mouse neural stem cell line C17.2 and Mouse primary NSCs	0.05–2 µM MeHg 24h or 48h for differentiation	↑Apoptosis (Bax activation; Cyt c translocation; Cas-3 activation and Calpain); Inhibition of NSC differentiation	[63]
8 weeks old C57BL/6 male mice Mouse neural stem C17.2 cell line	Single 25 mg/kg MeHg administration + 5 or 7 days free of MeHg; 5; 10 µM for 1-9h	↑TNF-α mRNA (cerebrum and cerebellum); ↓TNF-α mRNA;<br>↑ TNF-α release	[21]
Rat primary cortical neurons	0.25 -1µM MeHg for 6h	↓Cell viability; ↑Oxidative stress (↓SOD; ↓GSH; ↓GSH-Px); ↑Lipidic peroxidation	[64]
Swiss mice	4 mg/kg MeHg for 15 days	↓GSH and GST; ↑Lipidic peroxidation; ↑Mitochondrial damage; ↑Neuronal cell death in CA3 hippocampal region and PFC<br>↑Lipidic peroxidation	[65]
Rat primary cultures of CGCs	5-10 µM MeHg for 1h<br>1 µM for 18h	↑Necrosis (higher concentrations); ↑Apoptosis (lower + longer incubations)	[66]

Table S4: Biological models and MeHg exposure protocols used to evaluate neuron cellular alterations

Abbreviations:

Akt: Protein kinase B (PKB); AMPA: α-amino-3-hydroxy-5-methyl-4-isoxazolepropionic acid; KA: kainic acid; ATP: Adenosine-5'-triphosphate; Bax: bcl-2(B-cell lymphoma 2) -like protein 4; BDNF: Brain derived neurotrophic factor; CAT: Catalase; Cas-1: Caspase-1; Cas-3:Caspase-3; Cas-8:Caspase-8; CGC: cerebellar granule cells; CNTF: Ciliary neurotrophic factor; CSF: Cerebrospinal fluid; Cys: Cystein; Cyt c: Cytochrome c; DCX: Doublecortin; DG: Dentate Gyrus; ER: Endoplasmatic reticulum; ERK: Extracellular signal-regulated kinase; GCL: granule cell layer; GDNF: Glial cell-derived neurotrophic factor; GFAP: Glial fibrillary acidic protein; GLAST: Glutamate Aspartate Transporter 1; GLT-1: Glutamate transporter-1; GPx: Glutathione peroxidase; GS: Glutathione synthetase; GSH: Glutathione reduced; GSSG: Glutathione oxidant; Hg: Mercury HIF-1α: Hypoxia-inducible factor 1-alpha; hiPSC-MN: human induced pluripotent stem cell-derived motor neurons HO-1: Heme oxygenase-1; IFNγ: Interferon γ; IL-10: Interleukin-1;; IL-6: Interleukin-6; iNOS: Inducible nitric oxide synthase; JNK: c-Jun N-terminal kinases; LDH: Lactate dehydrogenase; MAP2: Microtubule-associated protein 2 ; MeHg: Methylmercury ; NAD+/NADH: Nicotinamide: adenine dinucleotide/ nicotinamide adenine dinucleotide reduced; NADPH: Nicotinamide adenine dinucleotide phosphate NeuN: Neuronal Nuclei; NF-H: Neurofilament – H; NF-kB: Nuclear factor kappa B; NMDA: N-methyl-D-aspartate receptor; NO: Nitric oxide; NPC: Neuronal progenitor cells; NQR: quinone oxidoreductase; Nrf2: Nuclear factor erythroid 2–related factor 2; NSC: Neuron Stem Cells; PARP: Poly (ADP-ribose) polymerase; PFC: Prefrontral cortex ; P-MYPT1: phospho-Myosin Phosphatase Target Subunit 1; P2Y1: Human purinergic G protein-coupled receptor; PI: Propidium iodide; PS: Phosphatidylserine; PSD-95: Postsynaptic density protein 95; P-STAT3: phospho-Signal transducer and activator of transcription 3 ; ROCK: Rho-associated protein kinase; ROS: Reactive Oxygen Species; S100β: S100 calcium-binding protein B; sci: subcutaneously injected; SNAP25: Synaptosomal-associated protein 25; SOD-1: Superoxide dismutase 1;SOX2: SRY (sex determining region Y)-box 2; SPP: Signal Peptide Peptidase ; TNF-α: Tumor necrosis factor α;

References

1. Nishioku, T.; Takai, N.; Miyamoto, K.; Murao, K.; Hara, C.; Yamamoto, K.; Nakanishi, H. Involvement of caspase 3-like protease in methylmercury-induced apoptosis of primary cultured rat cerebral microglia. *Brain Res* **2000**, *871*, 160-164, doi:10.1016/s0006-8993(00)02436-7.
2. Eskes, C.; Honegger, P.; Juillerat-Jeanneret, L.; Monnet-Tschudi, F. Microglial reaction induced by noncytotoxic methylmercury treatment leads to neuroprotection via interactions with astrocytes and IL-6 release. *Glia* **2002**, *37*, 43-52, doi:10.1002/glia.10019.
3. Garg, T.K.; Chang, J.Y. Methylmercury causes oxidative stress and cytotoxicity in microglia: attenuation by 15-deoxy-delta 12, 14-prostaglandin J2. *J Neuroimmunol* **2006**, *171*, 17-28, doi:10.1016/j.jneuroim.2005.09.007.
4. Tan, Q.; Zhang, M.; Geng, L.; Xia, Z.; Li, C.; Usman, M.; Du, Y.; Wei, L.; Bi, H. Hormesis of methylmercury-human serum albumin conjugate on N9 microglia via ERK/MAPKs and STAT3 signaling pathways. *Toxicol Appl Pharmacol* **2019**, *362*, 59-66, doi:10.1016/j.taap.2018.10.017.
5. Bassett, T.; Bach, P.; Chan, H.M. Effects of methylmercury on the secretion of pro-inflammatory cytokines from primary microglial cells and astrocytes. *Neurotoxicology* **2012**, *33*, 229-234, doi:10.1016/j.neuro.2011.10.003.
6. Ni, M.; Li, X.; Yin, Z.; Jiang, H.; Sidoryk-Wegrzynowicz, M.; Milatovic, D.; Cai, J.; Aschner, M. Methylmercury induces acute oxidative stress, altering Nrf2 protein level in primary microglial cells. *Toxicol Sci* **2010**, *116*, 590-603, doi:10.1093/toxsci/kfq126.

7. Ni, M.; Li, X.; Yin, Z.; Sidoryk-Wegrzynowicz, M.; Jiang, H.; Farina, M.; Rocha, J.B.; Syversen, T.; Aschner, M. Comparative study on the response of rat primary astrocytes and microglia to methylmercury toxicity. *Glia* **2011**, *59*, 810-820, doi:10.1002/glia.21153.
8. Monnet-Tschudi, F. Induction of apoptosis by mercury compounds depends on maturation and is not associated with microglial activation. *J Neurosci Res* **1998**, *53*, 361-367, doi:10.1002/(SICI)1097-4547(19980801)53:3<361::AID-JNR10>3.0.CO;2-8.
9. Chang, J.Y. Methylmercury causes glial IL-6 release. *Neurosci Lett* **2007**, *416*, 217-220, doi:10.1016/j.neulet.2007.01.076.
10. Shinozaki, Y.; Danjo, Y.; Koizumi, S. Microglial ROCK is essential for chronic methylmercury-induced neurodegeneration. *J Neurochem* **2019**, *151*, 64-78, doi:10.1111/jnc.14817.
11. Hoshi, T.; Toyama, T.; Shinozaki, Y.; Koizumi, S.; Lee, J.Y.; Naganuma, A.; Hwang, G.W. Evaluation of M1-microglial activation by neurotoxic metals using optimized organotypic cerebral slice cultures. *J Toxicol Sci* **2019**, *44*, 471-479, doi:10.2131/jts.44.471.
12. Sakamoto, M.; Miyamoto, K.; Wu, Z.; Nakanishi, H. Possible involvement of cathepsin B released by microglia in methylmercury-induced cerebellar pathological changes in the adult rat. *Neurosci Lett* **2008**, *442*, 292-296, doi:10.1016/j.neulet.2008.07.019.
13. Mariani, A.; Fanelli, R.; Re Depaolini, A.; De Paola, M. Decabrominated diphenyl ether and methylmercury impair fetal nervous system development in mice at documented human exposure levels. *Dev Neurobiol* **2015**, *75*, 23-38, doi:10.1002/dneu.22208.
14. Fujimura, M.; Usuki, F.; Nakamura, A. Fasudil, a Rho-Associated Coiled Coil-Forming Protein Kinase Inhibitor, Recovers Methylmercury-Induced Axonal Degeneration by Changing Microglial Phenotype in Rats. *Toxicol Sci* **2019**, *168*, 126-136, doi:10.1093/toxsci/kfy281.
15. Charleston, J.S.; Bolender, R.P.; Mottet, N.K.; Body, R.L.; Vahter, M.E.; Burbacher, T.M. Increases in the number of reactive glia in the visual cortex of Macaca fascicularis following subclinical long-term methyl mercury exposure. *Toxicol Appl Pharmacol* **1994**, *129*, 196-206, doi:10.1006/taap.1994.1244.
16. Charleston, J.S.; Body, R.L.; Mottet, N.K.; Vahter, M.E.; Burbacher, T.M. Autometallographic determination of inorganic mercury distribution in the cortex of the calcarine sulcus of the monkey Macaca fascicularis following long-term subclinical exposure to methylmercury and mercuric chloride. *Toxicol Appl Pharmacol* **1995**, *132*, 325-333, doi:10.1006/taap.1995.1114.
17. Monnet-Tschudi, F.; Zurich, M.G.; Honegger, P. Comparison of the developmental effects of two mercury compounds on glial cells and neurons in aggregate cultures of rat telencephalon. *Brain Res* **1996**, *741*, 52-59, doi:10.1016/s0006-8993(96)00895-5.
18. Yamamoto, M.; Takeya, M.; Ikeshima-Kataoka, H.; Yasui, M.; Kawasaki, Y.; Shiraishi, M.; Majima, E.; Shiraishi, S.; Uezono, Y.; Sasaki, M., et al. Increased expression of aquaporin-4 with methylmercury exposure in the brain of the common marmoset. *J Toxicol Sci* **2012**, *37*, 749-763, doi:10.2131/jts.37.749.
19. Fujimura, M.; Usuki, F.; Sawada, M.; Takashima, A. Methylmercury induces neuropathological changes with tau hyperphosphorylation mainly through the activation of the c-jun-N-terminal kinase pathway in the cerebral cortex, but not in the hippocampus of the mouse brain. *Neurotoxicology* **2009**, *30*, 1000-1007, doi:10.1016/j.neuro.2009.08.001.
20. Shinoda, Y.; Ehara, S.; Tatsumi, S.; Yoshida, E.; Takahashi, T.; Eto, K.; Kaji, T.; Fujiwara, Y. Methylmercury-induced neural degeneration in rat dorsal root ganglion is associated with the accumulation of microglia/macrophages and the proliferation of Schwann cells. *J Toxicol Sci* **2019**, *44*, 191-199, doi:10.2131/jts.44.191.

21. Iwai-Shimada, M.; Takahashi, T.; Kim, M.S.; Fujimura, M.; Ito, H.; Toyama, T.; Naganuma, A.; Hwang, G.W. Methylmercury induces the expression of TNF-alpha selectively in the brain of mice. *Sci Rep* **2016**, *6*, 38294, doi:10.1038/srep38294.
22. Shinozaki, Y.; Nomura, M.; Iwatsuki, K.; Moriyama, Y.; Gachet, C.; Koizumi, S. Microglia trigger astrocyte-mediated neuroprotection via purinergic gliotransmission. *Sci Rep* **2014**, *4*, 4329, doi:10.1038/srep04329.
23. Issa, Y.; Brunton, P.; Waters, C.M.; Watts, D.C. Cytotoxicity of metal ions to human oligodendroglial cells and human gingival fibroblasts assessed by mitochondrial dehydrogenase activity. *Dent Mater* **2008**, *24*, 281-287, doi:10.1016/j.dental.2007.09.010.
24. Pamphlett, R.; Kum Jew, S.; Doble, P.A.; Bishop, D.P. Elemental imaging shows mercury in cells of the human lateral and medial geniculate nuclei. *PLoS One* **2020**, *15*, e0231870, doi:10.1371/journal.pone.0231870.
25. Pamphlett, R.; Kum Jew, S. Inorganic mercury in human astrocytes, oligodendrocytes, corticomotoneurons and the locus ceruleus: implications for multiple sclerosis, neurodegenerative disorders and gliomas. *Biometals* **2018**, *31*, 807-819, doi:10.1007/s10534-018-0124-4.
26. Farina, M.; Cereser, V.; Portela, L.V.; Mendez, A.; Porciúncula, L.O.; Fornaguera, J.; Gonçalves, C.A.; Wofchuk, S.T.; Rocha, J.B.; Souza, D.O. Methylmercury increases S100B content in rat cerebrospinal fluid. *Environ Toxicol Pharmacol* **2005**, *19*, 249-253, doi:10.1016/j.etap.2004.07.008.
27. Ishihara, Y.; Itoh, K.; Oguro, A.; Chiba, Y.; Ueno, M.; Tsuji, M.; Vogel, C.F.A.; Yamazaki, T. Neuroprotective activation of astrocytes by methylmercury exposure in the inferior colliculus. *Sci Rep* **2019**, *9*, 13899, doi:10.1038/s41598-019-50377-9.
28. Liu, W.; Xu, Z.; Yang, T.; Deng, Y.; Xu, B.; Feng, S. Tea Polyphenols Protect Against Methylmercury-Induced Cell Injury in Rat Primary Cultured Astrocytes, Involvement of Oxidative Stress and Glutamate Uptake/Metabolism Disorders. *Mol Neurobiol* **2016**, *53*, 2995-3009, doi:10.1007/s12035-015-9161-y.
29. Yin, Z.; Lee, E.; Ni, M.; Jiang, H.; Milatovic, D.; Rongzhu, L.; Farina, M.; Rocha, J.B.; Aschner, M. Methylmercury-induced alterations in astrocyte functions are attenuated by ebselen. *Neurotoxicology* **2011**, *32*, 291-299, doi:10.1016/j.neuro.2011.01.004.
30. Yin, Z.; Milatovic, D.; Aschner, J.L.; Syversen, T.; Rocha, J.B.; Souza, D.O.; Sidoryk, M.; Albrecht, J.; Aschner, M. Methylmercury induces oxidative injury, alterations in permeability and glutamine transport in cultured astrocytes. *Brain Res* **2007**, *1131*, 1-10, doi:10.1016/j.brainres.2006.10.070.
31. Shanker, G.; Allen, J.W.; Mutkus, L.A.; Aschner, M. Methylmercury inhibits cysteine uptake in cultured primary astrocytes, but not in neurons. *Brain Res* **2001**, *914*, 159-165, doi:10.1016/s0006-8993(01)02791-3.
32. Chang, J.; Yang, B.; Zhou, Y.; Yin, C.; Liu, T.; Qian, H.; Xing, G.; Wang, S.; Li, F.; Zhang, Y., et al. Acute Methylmercury Exposure and the Hypoxia-Inducible Factor-1α Signaling Pathway under Normoxic Conditions in the Rat Brain and Astrocytes in Vitro. *Environ Health Perspect* **2019**, *127*, 127006, doi:10.1289/ehp5139.
33. Pierozan, P.; Biasibetti, H.; Schmitz, F.; Avila, H.; Fernandes, C.G.; Pessoa-Pureur, R.; Wyse, A.T.S. Neurotoxicity of Methylmercury in Isolated Astrocytes and Neurons: the Cytoskeleton as a Main Target. *Mol Neurobiol* **2017**, *54*, 5752-5767, doi:10.1007/s12035-016-0101-2.
34. Wang, L.; Jiang, H.; Yin, Z.; Aschner, M.; Cai, J. Methylmercury toxicity and Nrf2-dependent detoxification in astrocytes. *Toxicol Sci* **2009**, *107*, 135-143, doi:10.1093/toxsci/kfn201.
35. Yang, B.; Yin, C.; Zhou, Y.; Wang, Q.; Jiang, Y.; Bai, Y.; Qian, H.; Xing, G.; Wang, S.; Li, F., et al. Curcumin protects against methylmercury-induced cytotoxicity in primary rat

astrocytes by activating the Nrf2/ARE pathway independently of PKCδ. *Toxicology* **2019**, *425*, 152248, doi:10.1016/j.tox.2019.152248.
36. Kaur, P.; Aschner, M.; Syversen, T. Glutathione modulation influences methyl mercury induced neurotoxicity in primary cell cultures of neurons and astrocytes. *Neurotoxicology* **2006**, *27*, 492-500, doi:10.1016/j.neuro.2006.01.010.
37. Robitaille, S.; Mailloux, R.J.; Chan, H.M. Methylmercury alters glutathione homeostasis by inhibiting glutaredoxin 1 and enhancing glutathione biosynthesis in cultured human astrocytoma cells. *Toxicol Lett* **2016**, *256*, 1-10, doi:10.1016/j.toxlet.2016.05.013.
38. Dos Santos, A.A.; López-Granero, C.; Farina, M.; Rocha, J.B.T.; Bowman, A.B.; Aschner, M. Oxidative stress, caspase-3 activation and cleavage of ROCK-1 play an essential role in MeHg-induced cell death in primary astroglial cells. *Food Chem Toxicol* **2018**, *113*, 328-336, doi:10.1016/j.fct.2018.01.057.
39. Freire, M.A.M.; Lima, R.R.; Nascimento, P.C.; Gomes-Leal, W.; Pereira, A., Jr. Effects of methylmercury on the pattern of NADPH diaphorase expression and astrocytic activation in the rat. *Ecotoxicol Environ Saf* **2020**, *201*, 110799, doi:10.1016/j.ecoenv.2020.110799.
40. Burke, K.; Cheng, Y.; Li, B.; Petrov, A.; Joshi, P.; Berman, R.F.; Reuhl, K.R.; DiCicco-Bloom, E. Methylmercury elicits rapid inhibition of cell proliferation in the developing brain and decreases cell cycle regulator, cyclin E. *Neurotoxicology* **2006**, *27*, 970-981, doi:10.1016/j.neuro.2006.09.001.
41. Obiorah, M.; McCandlish, E.; Buckley, B.; DiCicco-Bloom, E. Hippocampal developmental vulnerability to methylmercury extends into prepubescence. *Front Neurosci* **2015**, *9*, 150, doi:10.3389/fnins.2015.00150.
42. Falluel-Morel, A.; Sokolowski, K.; Sisti, H.M.; Zhou, X.; Shors, T.J.; Dicicco-Bloom, E. Developmental mercury exposure elicits acute hippocampal cell death, reductions in neurogenesis, and severe learning deficits during puberty. *J Neurochem* **2007**, *103*, 1968-1981, doi:10.1111/j.1471-4159.2007.04882.x.
43. Sokolowski, K.; Obiorah, M.; Robinson, K.; McCandlish, E.; Buckley, B.; DiCicco-Bloom, E. Neural stem cell apoptosis after low-methylmercury exposures in postnatal hippocampus produce persistent cell loss and adolescent memory deficits. *Dev Neurobiol* **2013**, *73*, 936-949, doi:10.1002/dneu.22119.
44. Tian, J.; Luo, Y.; Chen, W.; Yang, S.; Wang, H.; Cui, J.; Lu, Z.; Lin, Y.; Bi, Y. MeHg Suppressed Neuronal Potency of Hippocampal NSCs Contributing to the Puberal Spatial Memory Deficits. *Biol Trace Elem Res* **2016**, *172*, 424-436, doi:10.1007/s12011-015-0609-8.
45. Jebbett, N.J.; Hamilton, J.W.; Rand, M.D.; Eckenstein, F. Low level methylmercury enhances CNTF-evoked STAT3 signaling and glial differentiation in cultured cortical progenitor cells. *Neurotoxicology* **2013**, *38*, 91-100, doi:10.1016/j.neuro.2013.06.008.
46. Bittencourt, L.O.; Dionizio, A.; Nascimento, P.C.; Puty, B.; Leão, L.K.R.; Luz, D.A.; Silva, M.C.F.; Amado, L.L.; Leite, A.; Buzalaf, M.R., et al. Proteomic approach underlying the hippocampal neurodegeneration caused by low doses of methylmercury after long-term exposure in adult rats. *Metallomics* **2019**, *11*, 390-403, doi:10.1039/c8mt00297e.
47. Freire, M.A.M.; Santana, L.N.S.; Bittencourt, L.O.; Nascimento, P.C.; Fernandes, R.M.; Leão, L.K.R.; Fernandes, L.M.P.; Silva, M.C.F.; Amado, L.L.; Gomes-Leal, W., et al. Methylmercury intoxication and cortical ischemia: Pre-clinical study of their comorbidity. *Ecotoxicol Environ Saf* **2019**, *174*, 557-565, doi:10.1016/j.ecoenv.2019.03.009.
48. Radio, N.M.; Freudenrich, T.M.; Robinette, B.L.; Crofton, K.M.; Mundy, W.R. Comparison of PC12 and cerebellar granule cell cultures for evaluating neurite outgrowth using high content analysis. *Neurotoxicol Teratol* **2010**, *32*, 25-35, doi:10.1016/j.ntt.2009.06.003.
49. Ferraro, L.; Tomasini, M.C.; Tanganelli, S.; Mazza, R.; Coluccia, A.; Carratù, M.R.; Gaetani, S.; Cuomo, V.; Antonelli, T. Developmental exposure to methylmercury elicits

early cell death in the cerebral cortex and long-term memory deficits in the rat. *Int J Dev Neurosci* **2009**, *27*, 165-174, doi:10.1016/j.ijdevneu.2008.11.004.
50. Fujimura, M.; Usuki, F.; Cheng, J.; Zhao, W. Prenatal low-dose methylmercury exposure impairs neurite outgrowth and synaptic protein expression and suppresses TrkA pathway activity and eEF1A1 expression in the rat cerebellum. *Toxicol Appl Pharmacol* **2016**, *298*, 1-8, doi:10.1016/j.taap.2016.03.002.
51. Fujimura, M.; Usuki, F. Methylmercury causes neuronal cell death through the suppression of the TrkA pathway: in vitro and in vivo effects of TrkA pathway activators. *Toxicol Appl Pharmacol* **2015**, *282*, 259-266, doi:10.1016/j.taap.2014.12.008.
52. Jacob, S.; Sumathi, T. Extenuation of in utero toxic effects of MeHg in the developing neurons by Fisetin via modulating the expression of synaptic transmission and plasticity regulators in hippocampus of the rat offspring. *Chem Biol Interact* **2019**, *305*, 3-10, doi:10.1016/j.cbi.2019.03.014.
53. Wang, X.; Yan, M.; Zhao, L.; Wu, Q.; Wu, C.; Chang, X.; Zhou, Z. Low-Dose Methylmercury-Induced Apoptosis and Mitochondrial DNA Mutation in Human Embryonic Neural Progenitor Cells. *Oxid Med Cell Longev* **2016**, *2016*, 5137042, doi:10.1155/2016/5137042.
54. Chung, Y.P.; Yen, C.C.; Tang, F.C.; Lee, K.I.; Liu, S.H.; Wu, C.C.; Hsieh, S.S.; Su, C.C.; Kuo, C.Y.; Chen, Y.W. Methylmercury exposure induces ROS/Akt inactivation-triggered endoplasmic reticulum stress-regulated neuronal cell apoptosis. *Toxicology* **2019**, *425*, 152245, doi:10.1016/j.tox.2019.152245.
55. Ke, T.; Tsatsakis, A.; Santamaría, A.; Antunes Soare, F.A.; Tinkov, A.A.; Docea, A.O.; Skalny, A.; Bowman, A.B.; Aschner, M. Chronic exposure to methylmercury induces puncta formation in cephalic dopaminergic neurons in Caenorhabditis elegans. *Neurotoxicology* **2020**, *77*, 105-113, doi:10.1016/j.neuro.2020.01.003.
56. Petroni, D.; Tsai, J.; Mondal, D.; George, W. Attenuation of low dose methylmercury and glutamate induced-cytotoxicity and tau phosphorylation by an N-methyl-D-aspartate antagonist in human neuroblastoma (SHSY5Y) cells. *Environ Toxicol* **2013**, *28*, 700-706, doi:10.1002/tox.20765.
57. Liu, W.; Wang, X.; Zhang, R.; Zhou, Y. Effects of postnatal exposure to methylmercury on spatial learning and memory and brain NMDA receptor mRNA expression in rats. *Toxicol Lett* **2009**, *188*, 230-235, doi:10.1016/j.toxlet.2009.04.021.
58. Colón-Rodríguez, A.; Colón-Carrión, N.M.; Atchison, W.D. AMPA receptor contribution to methylmercury-mediated alteration of intracellular Ca(2+) concentration in human induced pluripotent stem cell motor neurons. *Neurotoxicology* **2020**, *81*, 116-126, doi:10.1016/j.neuro.2020.09.037.
59. Sceniak, M.P.; Spitsbergen, J.B.; Sabo, S.L.; Yuan, Y.; Atchison, W.D. Acute neurotoxicant exposure induces hyperexcitability in mouse lumbar spinal motor neurons. *J Neurophysiol* **2020**, *123*, 1448-1459, doi:10.1152/jn.00775.2019.
60. Sakaue, M.; Maki, T.; Kaneko, T.; Hemmi, N.; Sekiguchi, H.; Horio, T.; Kadowaki, E.; Ozawa, A.; Yamamoto, M. Potentiation of Methylmercury-Induced Death in Rat Cerebellar Granular Neurons Occurs by Further Decrease of Total Intracellular GSH with BDNF via TrkB in Vitro. *Biol Pharm Bull* **2016**, *39*, 1047-1054, doi:10.1248/bpb.b16-00091.
61. Algarve, T.D.; Assmann, C.E.; Cadoná, F.C.; Machado, A.K.; Manica-Cattani, M.F.; Sato-Miyata, Y.; Asano, T.; Duarte, M.; Ribeiro, E.E.; Aigaki, T., et al. Guarana improves behavior and inflammatory alterations triggered by methylmercury exposure: an in vivo fruit fly and in vitro neural cells study. *Environ Sci Pollut Res Int* **2019**, *26*, 15069-15083, doi:10.1007/s11356-019-04881-0.
62. Fujimura, M.; Usuki, F. Methylmercury induces oxidative stress and subsequent neural hyperactivity leading to cell death through the p38 MAPK-CREB pathway in

differentiated SH-SY5Y cells. *Neurotoxicology* **2018**, *67*, 226-233, doi:10.1016/j.neuro.2018.06.008.
63. Tamm, C.; Duckworth, J.; Hermanson, O.; Ceccatelli, S. High susceptibility of neural stem cells to methylmercury toxicity: effects on cell survival and neuronal differentiation. *J Neurochem* **2006**, *97*, 69-78, doi:10.1111/j.1471-4159.2006.03718.x.
64. Liu, W.; Yang, T.; Xu, Z.; Xu, B.; Deng, Y. Methyl-mercury induces apoptosis through ROS-mediated endoplasmic reticulum stress and mitochondrial apoptosis pathways activation in rat cortical neurons. *Free Radic Res* **2019**, *53*, 26-44, doi:10.1080/10715762.2018.1546852.
65. Krishna Chandran, A.M.; Christina, H.; Das, S.; Mumbrekar, K.D.; Satish Rao, B.S. Neuroprotective role of naringenin against methylmercury induced cognitive impairment and mitochondrial damage in a mouse model. *Environ Toxicol Pharmacol* **2019**, *71*, 103224, doi:10.1016/j.etap.2019.103224.
66. Castoldi, A.F.; Barni, S.; Turin, I.; Gandini, C.; Manzo, L. Early acute necrosis, delayed apoptosis and cytoskeletal breakdown in cultured cerebellar granule neurons exposed to methylmercury. *J Neurosci Res* **2000**, *59*, 775-787, doi:10.1002/(sici)1097-4547(20000315)59:6<775::aid-jnr10>3.0.co;2-t.
